# How clinical practitioners assess frailty in their daily practice: an international survey

**DOI:** 10.1007/s40520-017-0806-8

**Published:** 2017-08-02

**Authors:** Olivier Bruyère, Fanny Buckinx, Charlotte Beaudart, Jean-Yves Reginster, Juergen Bauer, Tommy Cederholm, Antonio Cherubini, Cyrus Cooper, Alfonso Jose Cruz-Jentoft, Francesco Landi, Stefania Maggi, René Rizzoli, Avan Aihie Sayer, Cornel Sieber, Bruno Vellas, Matteo Cesari, on behalf of ESCEO and the EUGMS frailty working group

**Affiliations:** 1 Department of Public Health, Epidemiology and Health Economics, University of Liège, CHU Sart-Tilman, Bât B23, 4000 Liège, Belgium; 2 Center for Geriatric Medicine, Agaplesion Bethanien Hospital, University of Heidelberg, Heidelberg, Germany; 3 Department of Public Health and Caring Sciences, Clinical Nutrition and Metabolism, Uppsala University, Uppsala, Sweden; 4 Geriatrics and Geriatric Emergency Care, IRCCS-INRCA, Ancona, Italy; 5 MRC Lifecourse Epidemiology Unit, University of Southampton, Southampton, England UK; 6 Servicio de Geriatría, Hospital Universitario Ramón y Cajal (IRYCIS), Madrid, Spain; 7 Department of Geriatrics, Neurosciences and Orthopedics, Catholic University of the Sacred Heart School of Medicine, Rome, Italy; 8 National Research Council, Neuroscience Institute, Padua, Italy; 9 Geneva University Hospitals and Faculty of Medicine, Geneva, Switzerland; 10 NIHR Newcastle Biomedical Research Centre, Newcastle upon Tyne Hospitals NHS Foundation Trust and Faculty of Medical Sciences, Newcastle University, Newcastle upon Tyne, England UK; 11 Friedrich-Alexander-Universität Erlangen-Nürnberg, Nuremberg, Germany; 12 Gérontopôle de Toulouse, Département de Médecine Interne et Gérontologie Clinique, Centre Hospitalo-Universitaire de Toulouse, Toulouse, France

**Keywords:** Frailty, Survey, Clinical practice, Standardisation

## Abstract

**Introduction:**

Various operational definitions have been proposed to assess the frailty condition among older individuals. Our objective was to assess how practitioners measure the geriatric syndrome of frailty in their daily routine.

**Methods:**

An online survey was sent to national geriatric societies affiliated to the European Union Geriatric Medicine Society (EUGMS) and to members of the European Society for Clinical and Economic Aspects of Osteoporosis, Osteoarthritis and Musculoskeletal Diseases (ESCEO).

**Results:**

A total of 388 clinicians from 44 countries answered to the survey. Most of them were medical doctors (93%), and their primary field of practice was geriatrics (83%). Two hundred and five clinicians (52.8%) always assessed frailty in their daily practice, 38.1% reported to “sometimes” measure it, and 9.1% never assess it. A substantial proportion of clinicians (64.9%) diagnose frailty using more than one instrument. The most widely used tool was the gait speed test, adopted by 43.8% of the clinicians, followed by clinical frailty scale (34.3%), the SPPB test (30.2%), the frailty phenotype (26.8%) and the frailty index (16.8%).

**Conclusion:**

A variety of tools is used to assess frailty of older patients in clinical practice highlighting the need for standardisation and guidelines.

## Introduction

Frailty is a major syndrome associated with ageing [1]. This geriatric situation represents a huge potential public health issue at both the patient and the societal levels because of its multiple clinical, economic and societal consequences [2–5]. From a recent systematic review, it has been shown that frailty was associated with an increased risk of mortality, loss of activities in a normal daily routine, hospitalisation, physical limitations, falls and fractures [5].

Because of the high prevalence and the severity of such adverse outcomes, screening should be a priority, especially in primary care and taking advantage of any possible contact between the individual and the health care system [6–8]. Assessing frailty could indeed precociously identify persons at high risk of negative outcomes, theoretically allowing the timely implementation of preventive/therapeutic countermeasures.

Over the years, many models of frailty have been proposed. These models try to define the term frailty and construct a number of measurement tools to establish the frailty status of an individual. The most widely cited items on physical markers of frailty are based on the accumulation of deficits in physical, cognitive, mental health and functional domains. In a recent systematic review, 79 original or adapted frailty instruments were identified, but only 5 were linked to all 5 components of the International Classification of Functioning, Disability, and Health [9].

Major differences in the assessment of frailty exist in the clinical practice when taking care of older people [10]. Even the use of the same tools could be different, as recently highlighted in a study showing that geriatricians more often judge patients as frail compared to family physicians [11].

The variability in the operational definitions might be acceptable from a public health perspective, because the choice of an instrument should largely rely on the intervention to put eventually in place, the available resources and clinical priorities, factors that are extremely variable across settings and regions. At the same time, the identification of a gold standard measure might still be important to obtain because it represents the only way for providing to the construct of a nosological entity to the often ambiguous frailty condition [12].

To our knowledge, the frequency in the use of tools for the assessment of frailty and the components of this geriatric syndrome has never been estimated. Consequently, we designed an online survey with the objective to collect data looking at the proportion of clinicians assessing frailty in their daily practice and at the tools used by these clinicians.

## Materials and methods

An online survey using a self-administered questionnaire was designed to collect information on the tools used by clinicians for assessing frailty in their clinical practice. Responses to all questions were categorised but with the possibility to provide some open comments. The tools commonly used to assess frailty and then proposed in the questionnaire are presented in Table 1.

**Table 1:** Description of the tools commonly used to assess frailty

Tool used to assess frailty	Description of the tools
Gait speed [13]	To assess physical performances, gait speed, which is a component of the SPPB test, is also proposed by the EWGSOP. A score <0.8 m/s for walking speed is considered as poor physical performances
Clinical frailty scale [14]	This is based on a clinical evaluation in the domains of mobility, energy, physical activity and function, using descriptors and figures to stratify elderly adults according to their level of vulnerability. The score ranges from 1 (robust health) to 7 (complete functional dependence on others)
SPPB [13]	The short physical performance battery (SPPB) test is composed of three separate tests: balance, 4-metre gait speed and chair stand test. A score between 0 and 4 is assigned for each test, and the three tests are weighted equally. Therefore, the maximum score is 12 points. The cut-off value used to assess a poor physical performance is ≤8 points, according to the European working group on Sarcopenia in older people (EWGSOP)
Frailty phenotype [15] (i.e. fried criteria)	This is a deficit across five domains. Thus, phenotype of frailty was identified by the presence of three or more of the following components: shrinking, weakness, poor endurance and energy, slowness and a low level of physical activity. The presence of one or two deficits indicates a pre-frail condition, and a total of three or more deficits indicates frailty, while the absence of deficits indicates a robust state
Frailty index [16]	This is expressed as a ratio of deficits present to the total number of deficits considered. Frailty index includes 40 variables and the calculation was performed on the maximum number of deficits collected. Thus, participants were considered as frail when the ratio of deficits present to the total number of deficits considered was 0.25 (i.e. lowest quartile) or more
Edmonton frail scale [17]	This samples 8 domains (cognitive impairment, health attitudes, social support, medication use, nutrition, mood, continence, functional abilities). A score range between 0 and 3 is a robust state, 4–5 is a slightly frail state, 6–8 is a moderately frail state and 9–17 is a severely frail state
Frail scale status [18]	This has 5 components: fatigue, resistance, ambulation, illness and loss of weight. Scores range from 0 to 5 and represent frail (3–5), pre-frail (1–2) and robust (0) health states
Gerontopole frailty screening tool [19]	This is an 8-item questionnaire intended to help general practitioners identify frailty in community-dwelling persons 65 years or older without functional disability or current acute disease. The first 6 questions evaluate the patient’s status (living alone, involuntary weight loss, fatigue, mobility difficulties, memory problems and gait speed), whereas the last two assess the general practitioner’s personal view about the frailty status of the individual and the patient’s willingness to be referred to the Frailty Clinical for further evaluation
SHARE frailty instrument [20]	Using the five SHARE frailty variables (fatigue, loss of appetite, grip strength, functional difficulties and physical activity), D-factor scores (DFS) were determined using the SHARE-FI formula, and based on the DFS value, the subject could then be categorised as non-frail, pre-frail or frail
SEGA grid [21]	This establishes a risk profile of frailty and provides reporting of problems and factors that may influence functional decline, including age, provenance, drugs, mood, perceived health, history of falls, nutrition, comorbidities, IADL, mobility, continence, feeding and cognitive functions. A score of 0, 1 or 2 is given for each item and a total over 11 points indicates a “very frail” condition; a score between 8 and 11 points indicates a frail condition, while a score below 8 is a slightly frail condition
Groningen frailty indicator [22]	This consists of 15 self-report items and screens for loss of functions and resources in four domains: physical, cognitive, social, and psychological. Scores range from zero (not frail) to fifteen (very frail). A GFI score of 4 or higher was regarded as frail
Strawbridge questionnaire [23]	This defines frailty as difficulty in two or more functional domains (physical, cognitive, sensory and nutritive). A score greater than or equal to 3 in more than one domain is considered vulnerable
Tilburg frailty indicator [24]	It consists of 2 parts. Part A contains 10 questions on determinants of frailty and diseases (multimorbidity); part B contains 3 domains of frailty (quality of life, disability and health care utilisation) with a total of 15 questions on components of frailty. The threshold above which the participant is considered as frail is 5 points

The reasons why clinicians assessed frailty in their practice were also collected using predefined options. The clinician was specifically asked to consider his/her clinical practice directed towards subjects aged 60 years and older. Moreover, questions related to the assessment of domains traditionally considered as constituent of frailty (i.e. physical performance, nutritional status, cognitive status, biological markers, quality of life, depression, sensorial impairment and body composition) were also asked, using predefined categories, with the possibility to provide open answers. The same technique was used to collect the socio-demographic information of the clinician (e.g. country of residence, age, work place, profession).The time to complete the survey was around 5 min.

The survey was sent in January 2016 through two different channels. The first was the European Union Geriatric Medicine Society (EUGMS) office that forwarded the survey to all their affiliated societies who then forwarded it to their individual members. The second was a direct contact, via email, to all members of the European Society for Clinical and Economic Aspects of Osteoporosis, Osteoarthritis and Musculoskeletal Disorders (ESCEO).

Quantitative variables were expressed as means and standard deviations (SD) and qualitative variables as numbers and percentages. Analyses of variances were used to compare the prevalence of frailty assessment among different groups of clinicians. Results were considered to be statistically significant at the 5% critical level (*p* < 0.05). All calculations were performed using Statistica 10 software.

## Results

Three hundred and eighty-eight clinicians from 44 countries answered the survey. Most of the answers were obtained from Italy, Spain, Belgium and the United Kingdom (72.4% in total). Geriatrics was the most widely cited field of interest (83.6%), followed by rheumatology (6.6%) and endocrinology (3.4%). The vast majority of the clinicians were medical doctors (88.7%). Clinicians were on average 48.7 ± 12.1 years old, and 51.8% of them were men.

Two hundred and five clinicians (52.8%) always assessed frailty in their daily practice, 38.1% reported to “sometimes” measure it, and 9.1% never assess it. Consequently, frailty was assessed in the routine practice by 90.9% of the respondents. As expected, the frequency of the frailty assessment was higher among geriatricians (94.9%) compared with other specialities (75.4%; *p* < 0.0001). The most widely reported tool used to assess frailty was the gait speed (43.8%), followed by the clinical frailty scale (34.3%), the SPPB test (30.2%) and the Frailty Phenotype also known as the Fried Criteria (26.8%; Table 2). Among clinicians who assessed frailty with the frailty phenotype (i.e. 26.8%), 48.2% of them used the original version; 32.1% used an adapted version, whereas 19.7% did not know which version they used. It is important to note that 41.2% of clinicians assess frailty with a tool that was not proposed in the survey, and that 64.9% diagnose frailty using more than one instrument. The gait speed test and the SPPB are widely used in combination with another tool to assess frailty (in 89.3 and 73.3% of cases, respectively). These tools included LASA Physical Activity Questionnaire, the Physical Activity Scale for the Elderly (PASE), the Health Assessment Questionnaire (HAQ), the Short QUestionnaire to ASsess Health-enhancing physical activity (SQUASH), clinical history or self-made questionnaire.

**Table 2:** Tools used to assess frailty in clinical practice

Tool used	Number	Frequency (%)
Gait speed	170	43.8
Clinical frailty scale	133	34.3
SPPB	117	30.2
Frailty phenotype (i.e. Fried criteria)	104	26.8
Frailty index	65	16.8
Frail scale status	47	12.1
Edmonton frail scale	36	9.28
Gerontopole frailty screening tool	28	7.22
SHARE frailty instrument	16	4.12
SEGA grid	15	3.87
Groningen frailty indicator	10	2.55
Strawbridge questionnaire	8	2.06
Tilburg frailty indicator	5	1.29
Other	160	41.2

It is interesting to note that a similar pattern is observed in European countries and non-European ones. The most used tool was in both cases the gait speed (42.9% in Europe and 54.8% in the rest of the world). The Clinical Frailty Scale was used by 34.4% of the clinicians in Europe and by 33.3% out of Europe. The SPPB test was used by, respectively, 30.6 and 26.2% of the clinicians in Europe and out of Europe. Our survey also highlighted that clinicians working in a University or a University Hospital were not more likely to use frailty assessment tools than their counterparts working in general hospitals.

Among the most widely adopted tools, a specific cut-off was used by 35.3% of the clinicians for gait speed and by 24.8% of the clinicians for the SPPB. Almost half of the clinicians using the SPPB in their clinical practice (44.8%) used the 8-point cut-off. Among those who assessed gait speed in their clinical practice, 64.9% used the cut-off of 0.8 m/s to diagnose frailty.

The most frequently cited reasons why clinicians always assess frailty in their clinical practice were (1) frail older people are at high risk of adverse outcomes (i.e. falls, hospitalisations and death), and (2) its presence may affect the clinical decision. It is noteworthy that 71.6% of the clinicians always assess frailty because of a combination of several factors. The combination “because it is recommended to measure frailty among older people” AND “because the prevalence of frailty is high” AND “because frail older people are at high risk of falls, hospitalisations and death” AND “because its presence may affect the clinical decision” was the most frequently reported combination, reported by 13 clinicians (6.84%) (Table 3).

**Table 3:** Main reasons given by clinicians for always assessing frailty (*n* = 205)

Reasons	Number	Frequency (%)
Because frail older people are at high risk of falls, hospitalisations, death	21	10.3
Because its presence may affect my clinical decision	19	9.25
Because the prevalence of frailty is high	8	3.90
Because it is recommended to measure frailty among older people	6	2.93
Other	15	7.32
Combination of several factors	136	66.3

Among those who “sometimes” assess frailty, the most frequent reasons for conducting the evaluation are that (1) the patient seems frail (12.2%) and (2) the clinician may change his clinical decision according to the results of the test (7.43%). Around 63% of the clinicians assess frailty when several combined factors are observed, mainly when “the patient seems frail” AND “the patient has a low BMI (<21 kg/m²)” AND “the patient seems under-nourished” (2.03%) (Table 4).

**Table 4:** Main reasons given by clinicians for sometimes assessing frailty (*n* = 148)

Reasons	Number	Frequency (%)
When the patient seems frail	18	12.2
When I may change my clinical decision according to the result of the test	11	7.43
When I have time	10	6.76
When I think about it	4	2.70
Other	11	7.43
Combination of several factors	94	63.51

Lastly, the main reasons for never evaluating frailty (*n* = 35) are because clinicians have no time (12.5%), they think that there are no appropriate tools to assess frailty in clinical practice (9.38%), the results of such assessment do not substantially change the clinical decision (9.38%), clinicians do not feel competent to diagnose frailty (9.38%) and for a combination of several factors (*n* = 59.36%).

Besides the specific tools used in the diagnosis of frailty, other assessments that help to have a better view of the frailty status of the patient were performed by the clinicians (Table 5). They could be used as complementary tools after the operational diagnosis of frailty or for the general assessment of health status in the absence of a formal diagnosis of frailty. Functional status was measured by 79.4% of the clinicians, autonomy by 82.7%, nutritional status by 79.9%, cognitive status by 90.9%, sensorial impairment by 40.5%, biological markers by 60.1%, body composition by 40.5% and quality of life by 37.1%. However, among clinicians assessing at least one of these components of frailty, the tools used are different even if some particular tools are more widely used than others (e.g. MMSE and MNA).

**Table 5:** Assessment of frailty components in addition or in the absence of an operational diagnosis of frailty

Frailty component	Specific tool	Number	Frequency (%)
Functional status			
	SPPB test	158	40.7
	Gait speed	217	55.9
	Grip strength	158	40.7
	Other	39	10.1
Nutritional status			
	MNA (mini nutritional assessment)	221	56.9
	MUST (malnutrition universal screening tool)	42	10.8
	NRS (nutrition risk screening)	20	5.15
Cognitive status			
	MMSE (mini mental state examination)	297	76.5
	CRD (clinical dementia rating scale)	105	27.1
	FCSRT (free and cued selective reminding test)	7	1.8
	Executive function (i.e. memory, anxiety, attention)	84	21.6
	GDS (geriatric depression scale)	257	66.2
	Raskin depression scale	7	1.8
	Covi anxiety scale	5	1.29
	NPI scale	114	29.4
Autonomy			
	ADL (activity daily living)	261	67.3
	IADL (instrumental activity daily living)	246	63.4
	Other	43	11.1
Sensorial impairment			
	Sensorial	114	29.4
	Vision	157	40.5
	Monoyer-parinaud scale	26	6.7
	Amsler scale	25	6.4
	Audition	109	28.1
	HHIES (hearing handicap inventory for the elderly)	27	6.9
Biological markers			
	IL-6	20	5.15
	IGF-1	21	5.41
	Vitamin D	213	54.9
Body composition			
	BIA (bioelectrical impedance analysis)	72	18.6
	DXA (dual-energy X-rays absorptiometry)	64	16.5
	CT scan	19	4.89
	MRI (magnetic resonance imaging)	15	3.87
	Anthropometric values	75	19.3
Level of physical activity			
	Questionnaire	62	15.9
	Physical exhaustion or early fatigability	157	40.5
	Objective measurement	73	18.8
Quality of life		144	37.1
Socio-demographic data		292	75.3

## Discussion

The results of this survey highlight that multiple tools are used by clinicians to assess frailty. Moreover, even if the same tool might be used, a heterogeneity about the choice of the thresholds of risk also exists, as shown for the gait speed and the SPPB test. It is also important to note that in the absence of a consensual and operational definition of frailty, a lot of tools are used by the clinician for the general assessment of the health status of the subject aged over 65 years even in the absence of a formal diagnosis of frailty.

Interestingly, several frailty measurements used had not been robustly validated in the literature, and their prognostic ability was rarely determined. Moreover, many were modified from their original, validated version, potentially impacting on the replicability and comparability of the findings. Consistently with Theou and colleagues who reported the existence of 262 different versions of frailty phenotype [25], 32.1% of the respondents to our survey declared using a modified version of this operational definition.

It has been suggested, in the literature, that some of the tools used by clinicians are better for population-level frailty screening, whereas others are best suited for clinical screening, or for clinical assessment. Even if it was not the objective of the present survey, we believe it is also important to underline the difference between the concept of screening and the concept of diagnosis. The primary purpose of screening tests is to detect risk factors for disease in large numbers of apparently healthy individuals. The purpose of a diagnostic test is to establish the presence of disease in symptomatic or screening-positive individuals. Only the latter point is a basis for treatment decisions. Consequently, the tools to be used should be differentiated according to the purpose of the assessment [26]. This is probably a limitation of the present survey since we did not specifically ask whether the assessment of frailty was conducted for screening or diagnosing.

Currently, various tools are proposed to assess frailty but none has been able to prevail as superior to the others. The ideal scoring system for defining frailty should be able to assess all domains of frailty and their severity, as well as to be easily applicable to the daily clinical practice in order to measure changes occurring over time (especially after significant medical or surgical interventions) [27]. However, because of the multifactorial nature of frailty, it is difficult to construct a model that is both highly accurate and easy to use. However, it should be acknowledged that the psychometric properties requested for a screening or a diagnosis tool are not similar, a high accuracy for the diagnosis instrument and a fast and easy use for the screening tool.

According to a systematic review, the main factors associated with frailty are age, female gender, ethnic group, education, income, cardiovascular diseases, number of comorbidities, functional incapacity, poor self-rated health, depressive symptoms, cognitive function, body mass index, smoking and alcohol use [28]. Most of this information is routinely collected by clinicians. Interestingly, the cognitive domain of frailty is largely assessed with more than 90% of clinicians taking it into account. This is important because there is increasing evidence that cognitive decline synergistically acts with physical frailty to accelerate the trajectory towards disability [29–33]. However, the tools used by the clinicians to assess cognitive function are quite heterogeneous as well.

Even if this study had primarily a European perspective, we received some information from clinicians living outside Europe. Because of the few responses received, comparative analysis could not be performed. However, looking at the literature from developing countries, the frailty phenotype is widely diffused although the Frailty Index and the Edmonton Frailty Scale are also commonly used [34, 35]. In regions where health care resources are scarce, many health conditions frequently remain undiagnosed or are overlooked. Moreover, the lack of equipment and/or trained staff is another possible issue affecting the frailty assessment [34]. Moreover, it should be acknowledged that very few assessment tools have been fully validated in low- and medium-income countries.

Even in developed countries, different strategies and tools to assess frailty might be needed according to the clinical setting [36]. Given the magnitude of the challenges that frailty poses for the health care systems as currently organised, policy changes will be essential. To systematically implement the frailty assessment (i.e. as screening or as diagnosis), a number of policy changes are required, as proposed by the Canadian frailty network [36]. A common language needs to be used among researchers, health care providers, administrators, policy makers and the public, particularly in the definition of frailty. Researchers and clinicians need to identify and agree on which validated frailty tools to use in which setting. Before the full diagnosis of frailty by the clinician specialist, a consensus is needed on whom to screen. Possible approaches include screening all individuals over a certain age who come into contact with the health care system, or screening on the basis of selected criteria such as age, selected medical conditions, psychosocial disorders, falls or functional disability, high use of the health care system and change in living situation such as moving from independent to assisted accommodation.

In conclusion, frailty (in its physical and cognitive components) is regularly assessed by clinicians working with older subjects, using different standardised tools. A better understanding of the prognostic characteristics of such tools is needed to inform clinicians about these choices.
